# Effects of Virtual Reality on Pain, Anxiety, and Fear During Thyroid Fine-Needle Aspiration Biopsy: Open-Label Randomized Controlled Trial

**DOI:** 10.2196/103829

**Published:** 2026-10-06

**Authors:** Emine Karadeniz, Hamdiye Arda, Esma Gülsun Arslan Cellat

**Affiliations:** 1 Emergency Department Abdulkadir Yüksel State Hospital, Gaziantep Gaziantep, East Turkey Turkey; 2 Department of Nursing Atatürk Faculty of Health Sciences Dicle University Diyarbakır, East Turkey Turkey; 3 Department of Endocrinology and Metabolic Diseases Endocrine and Metabolic Diseases Specialist Clinic Gaziantep, East Turkey Turkey

**Keywords:** anxiety, fear, FNAB, pain, thyroid cancer suspicion, thyroid fine-needle aspiration biopsy, virtual reality, VR

## Abstract

**Background:**

Thyroid fine-needle aspiration biopsy (FNAB) is a commonly used diagnostic procedure in patients with suspected thyroid cancer; however, it may induce pain, anxiety, and fear during the procedure.

**Objective:**

This open-label randomized controlled trial aimed to evaluate the effect of virtual reality (VR) on pain as the primary outcome and anxiety and fear of pain as secondary outcomes in patients undergoing thyroid FNAB.

**Methods:**

The study was conducted between January 19, 2025, and April 30, 2025, at Gaziantep City Hospital, Türkiye. A total of 100 patients with suspected thyroid nodules were randomly assigned to either a VR intervention group (n=50) or a control group (n=50). Data were collected using a patient information form, the visual analog scale (VAS), the Beck Anxiety Inventory (BAI), and the Fear of Pain Questionnaire-III (FPQ-III).

**Results:**

After adjustment for baseline pain, previous thyroid mass diagnosis, and voice tone changes, the VR group had statistically significantly lower postintervention pain scores than the control group (adjusted mean 3.627 vs 4.493; *F*_1,95_=4.021; *P*=.048; partial η^2^=0.041). However, the unadjusted between-group comparison for pain was not statistically significant (*P*=.12), and the unadjusted effect size was small, with a 95% CI that crossed 0 (Cohen *d*=−0.33, 95% CI −0.72 to 0.07). No statistically significant adjusted between-group differences were observed for anxiety (*P*=.48) or fear of pain (*P*=.07). Unadjusted standardized between-group effect sizes were also small for anxiety (*d*=−0.17) and fear of pain (*d*=−0.11).

**Conclusions:**

The adjusted analysis suggested a small reduction in procedural pain with VR; however, the between-group difference was not statistically significant in the unadjusted analysis and reached statistical significance only after adjustment for baseline pain and 2 nonprespecified covariates selected on the basis of observed baseline imbalance. Moreover, the observed adjusted effect (f=0.207) was smaller than the minimum effect size the trial was powered to detect (f=0.283). No statistically significant adjusted between-group effects were found for anxiety or fear of pain. Therefore, the potential analgesic effect of VR should be interpreted cautiously and confirmed in larger, adequately powered trials.

**Trial Registration:**

ClinicalTrials.gov NCT06792929; https://clinicaltrials.gov/study/NCT06792929

## Introduction

### Background

Thyroid cancer is one of the malignancies whose incidence has increased worldwide in recent years and constitutes an important public health problem. Global epidemiological data indicate that the incidence of thyroid cancer has increased significantly over the last 3 decades [1]. In the United States, thyroid cancer incidence increased substantially over previous decades; however, the rate of increase slowed considerably after 2009 and subsequently reached a plateau, with incidence remaining stable or showing a declining trend after approximately 2014 [1,2]. In contrast, the mortality rate associated with thyroid cancer is quite low compared with that of many other malignancies, with an annual death rate of approximately 0.5 per 100,000 individuals [3]. This situation highlights the importance of early diagnosis and appropriate treatment strategies.

The etiology of thyroid cancer is multifactorial and is associated with various risk factors such as genetic predisposition, environmental factors, iodine intake level, hormonal factors, and particularly exposure to ionizing radiation during childhood [4]. Thyroid nodules are quite common in the general population and can be detected by palpation in approximately 4% to 8% of individuals, whereas this rate may increase to 40% to 50% when high-resolution ultrasonography is used [5,6]. Therefore, the evaluation of thyroid nodules in terms of malignancy plays an important role in clinical practice.

Clinical history, physical examination, laboratory tests, and imaging methods are used in the evaluation of thyroid nodules. Thyroid ultrasonography is a noninvasive and reliable diagnostic tool that provides detailed information about the size, structure, echogenicity, and vascularity of the nodule [7]. However, the definitive diagnosis of nodules suspected of malignancy is most commonly established by thyroid fine-needle aspiration biopsy (FNAB). FNAB is considered one of the gold standard methods for the evaluation of thyroid nodules due to its high diagnostic accuracy, low complication rate, and cost-effectiveness [8,9].

Although FNAB is generally considered a minimally invasive and well-tolerated procedure, some patients may experience pain and anxiety associated with the procedure [10-12]. Leboulleux et al [11] reported that a proportion of patients experience clinically significant pain after thyroid FNAB, defined as a visual analog scale (VAS) score greater than or equal to 30 mm, and demonstrated that higher anxiety levels were significantly associated with greater pain intensity. Similarly, recent evidence has shown that preprocedural anxiety and pain perception are clinically relevant factors in patients undergoing thyroid FNAB [10], while a literature review has identified multiple patient-related and procedure-related factors that may influence pain experienced during thyroid FNAB [12]. Taken together, these findings indicate that, despite the brief and minimally invasive nature of FNAB, procedure-related pain and anxiety may represent relevant concerns for some patients, supporting the evaluation of nonpharmacological interventions aimed at improving patient comfort during the procedure [9-12].

In recent years, interest in nonpharmacological approaches to pain and anxiety management in health care has increased. Nurses use various nonpharmacological methods such as distraction, relaxation techniques, massage, meditation, and cognitive interventions to reduce pain, fear, and anxiety in patient care [13]. One of these methods, virtual reality (VR) technology, is an innovative approach that enables users to cognitively distance themselves from the real environment by directing their attention to a virtual environment [14].

VR technology provides an immersive experience by stimulating individuals’ visual and auditory senses through computer-assisted 3D environments. In this way, the individual’s focus of attention is diverted away from the stimulus causing pain, thereby reducing perceived pain and anxiety levels [15]. In recent years, the use of VR applications in the health care field has gradually increased, and it has been shown to be effective in the management of pain and anxiety, particularly during interventional procedures.

For example, a randomized controlled trial conducted during peripheral intravenous catheter placement in pediatric patients found that immersive VR significantly reduced pain and anxiety [16]. Similarly, a systematic review and meta-analysis demonstrated beneficial effects of VR on pain and anxiety during various gynecological procedures [17]. However, findings have not been consistent across all procedural settings. In a randomized controlled trial of patients undergoing ultrasound-guided breast biopsy, VR was associated with reduced anxiety, whereas no significant between-group difference in pain was observed [18]. Collectively, this evidence suggests that the effects of VR may vary according to the type of procedure and outcome assessed. Although VR has shown potential as a distraction method during invasive and minimally invasive procedures, evidence regarding its effects on pain, anxiety, and fear of pain during thyroid FNAB remains limited. In this context, the aim of this open-label randomized controlled trial was to evaluate the effects of VR on pain as the primary outcome and anxiety and fear of pain as secondary outcomes in patients undergoing thyroid FNAB for suspected thyroid cancer.

### Research Hypotheses

#### Primary Hypothesis

Hypothesis 1 was that patients receiving the VR intervention would report significantly lower pain (measured by the VAS) during thyroid FNAB than those receiving standard care.

#### Secondary Hypotheses

Hypothesis 2 was that patients receiving the VR intervention would report significantly lower anxiety (measured by the Beck Anxiety Inventory [BAI]) during thyroid FNAB than those receiving standard care. Hypothesis 3 was that patients receiving the VR intervention would report significantly lower fear of pain (measured by the Fear of Pain Questionnaire-III [FPQ-III]) during thyroid FNAB than those receiving standard care.

## Methods

### Study Design

This study was designed as an open-label randomized controlled trial with blinded statistical analysis. Statistical analyses were performed by an independent statistician who was blinded to group allocation.

### Setting and Time

The study was conducted in the endocrinology outpatient clinic of Gaziantep City Hospital in Gaziantep, Türkiye, between January 19, 2025, and April 30, 2025. Patients with suspected thyroid nodules are evaluated in the biopsy unit of this clinic, where approximately 170 thyroid FNAB procedures are performed each month across all physicians. The clinic comprises 3 examination rooms, 1 ultrasonography room, and 1 biopsy room and is staffed by 4 specialist physicians, 4 clinical nurses, and 5 medical secretaries.

### Population and Sample

The study population consisted of patients presenting to Gaziantep City Hospital with suspected thyroid nodules requiring FNAB. To enhance procedural consistency and reduce variability associated with differences in biopsy technique, recruitment was restricted to patients scheduled to undergo FNAB by 1 designated specialist physician. Potentially eligible patients were screened only on days when the researcher was present in the unit; therefore, recruitment was not consecutive across all patients undergoing FNAB during the study period. Within this recruitment framework, 110 patients were assessed for eligibility, and 100 eligible and consenting patients were enrolled and randomized.

### Inclusion and Exclusion Criteria

Participants were eligible for inclusion if they were aged 18 years or older, had a suspected thyroid nodule requiring biopsy, could speak and understand Turkish, had no cognitive impairments, had no prior experience with VR devices, and voluntarily agreed to participate, while those who did not volunteer, could not communicate in Turkish, or had vertigo, visual or hearing impairments, or cognitive disabilities that could interfere with the intervention were excluded.

### Sample Size and Power Analysis

The initial sample size calculation was performed a priori using G*Power based on a 2-tailed independent-samples *t* test, with an assumed effect size of *d*=0.50, a 2-sided α level of .05, and 80% power [19]. However, because the primary inferential analysis in the present study was analysis of covariance (ANCOVA) rather than an independent-samples *t* test, an additional sensitivity analysis aligned with the final ANCOVA model was conducted during manuscript revision. For this analysis, the final sample size of 100 participants (50 per group), a 2-sided α level of .05, 80% power, 2 groups, 1 numerator degree of freedom, and 3 covariates were specified. The sensitivity analysis indicated a minimum detectable effect size of f=0.283. Thus, with the final sample size, the study had 80% power to detect approximately moderate adjusted group effects, whereas smaller effects may not have been detected reliably.

### Randomization and Blinding

After obtaining ethical approval, gender-stratified randomization was used to achieve balanced allocation between the intervention and control groups. Separate randomization sequences were prepared for male and female participants before recruitment. After eligibility had been confirmed and written informed consent had been obtained, enrolled participants were assigned sequentially according to the preprepared gender-stratified randomization list. No formal allocation concealment mechanism, such as sequentially numbered opaque sealed envelopes or centralized allocation, was used.

The study was conducted as an open-label randomized controlled trial. Due to the nature of the intervention, blinding of participants and health care providers was not feasible. However, the independent statistician responsible for data analysis was blinded to group allocation. Group assignments were managed by a researcher who was not involved in data collection or statistical analysis.

The study information was first submitted to ClinicalTrials.gov (NCT06792929) on January 18, 2025, before enrollment of the first randomized participant on January 19, 2025, and the record was publicly posted on January 27, 2025, following registry processing. A separate preparatory pilot conducted in October 2024 involved 5 individuals who were neither randomized nor included in the trial analyses. The registry record was subsequently updated to “completed,” with an actual enrollment of 100 participants, an actual study start date of January 19, 2025, and actual primary and study completion dates of April 30, 2025.

### Participant Recruitment and Randomization

A total of 110 patients were assessed for eligibility within the defined recruitment framework described above. A total of 6 individuals did not meet the inclusion criteria, and 4 declined to participate. Consequently, 100 participants were enrolled and randomized. Detailed information regarding participant recruitment, allocation, and follow-up is presented in the CONSORT (Consolidated Standards of Reporting Trials) flow diagram (Figure 1). The CONSORT-EHEALTH (Consolidated Standards of Reporting Trials of Electronic and Mobile Health Applications and Online Telehealth) checklist is available in Multimedia Appendix 1.

**Figure 1:**
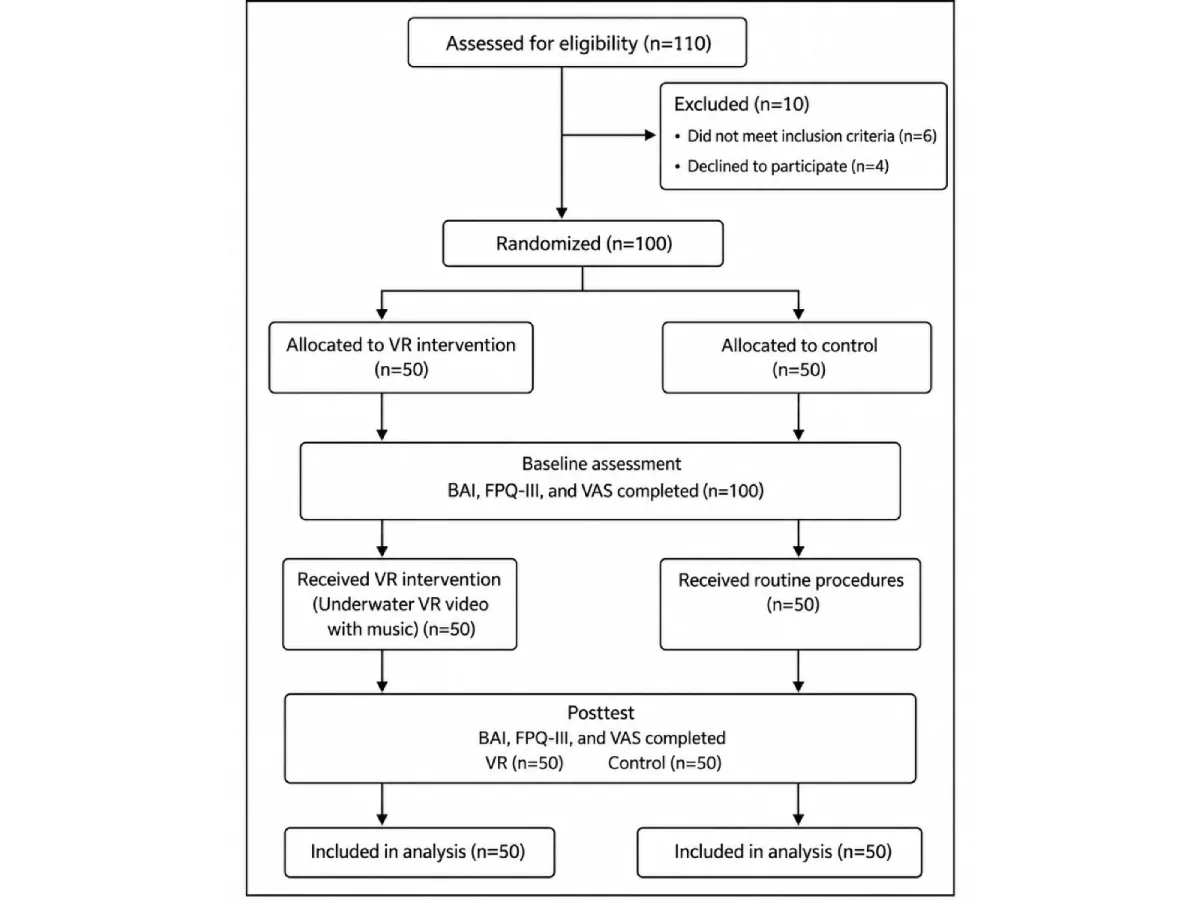
CONSORT (Consolidated Standards of Reporting Trials) flow diagram showing the recruitment, randomization, and analysis of participants.

### Data Collection Tools

#### Overview

Data were collected using the patient information form developed by the researchers, the VAS, the BAI, and the FPQ-III. The primary outcome was pain measured using the VAS, whereas the secondary outcomes were anxiety measured using the BAI and fear of pain measured using the FPQ-III.

#### Patient Information Form

The patient information form was developed by the researchers based on the relevant literature [1-18]. The form consists of 20 questions assessing sociodemographic characteristics (age, gender, and educational level), smoking and alcohol use, exercise habits, regular medication use, history of thyroid nodules, family history of thyroid nodules, presence of neck swelling, difficulty swallowing or breathing, sensation of throat pressure, voice changes, weight changes, fatigue, previous thyroid surgery, presence of chronic disease, previous experience with VR devices, and fear of FNAB.

#### VAS

The VAS (developed by Price et al [20]) is a widely used method for subjective pain assessment. The scale consists of a 10-cm line with end points labeled “no pain” and “worst imaginable pain.” Participants indicate their perceived pain level on the line, where 0 represents no pain and 10 represents the most severe pain. Higher scores indicate greater pain intensity. The VAS has been widely validated and adapted for use in clinical and research settings globally.

#### BAI

The BAI (developed by Beck et al [21]) is used to assess the severity of anxiety symptoms. The Turkish validity and reliability study was conducted by Ulusoy et al [22]. The scale consists of 21 items rated on a 4-point scale ranging from 0 to 3, with total scores ranging from 0 to 63. Higher scores indicate greater anxiety severity. Ulusoy et al [22] reported a Cronbach α coefficient of 0.93 for the Turkish version. In the present study, the Cronbach α was 0.86.

#### FPQ-III

The FPQ-III (developed by McNeil and Rainwater [23]) is used to evaluate fear related to painful experiences. The scale consists of 30 items, divided into 3 subscales: severe pain fear, minor pain fear, and medical pain fear. Each item is rated on a 5-point Likert scale ranging from 1 (not at all) to 5 (extreme), with total scores ranging from 30 to 150. Higher scores indicate greater fear of pain. The Turkish validity and reliability study was conducted by Ünver and Turan [24], reporting a Cronbach α of 0.93. In the present study, reliability coefficients were 0.87 for severe pain fear, 0.83 for minor pain fear, 0.86 for medical pain fear, and 0.93 for the total scale.

### Data Collection Procedure

Data were collected through face-to-face interviews conducted by the researcher. Participants completed the questionnaires in approximately 15 minutes.

Prior to the main study, the intervention procedures were pilot-tested with 5 patients to evaluate the feasibility and acceptability of the VR application, including headset use, questionnaire administration, and the overall study workflow. No modifications to the intervention protocol were required following pilot testing, and data obtained from the pilot participants were not included in the final analyses.

For the VR group, preprocedure questionnaires were administered 15 minutes before the biopsy procedure. Participants then underwent the biopsy while wearing the VR headset. The duration of VR exposure varied according to the biopsy procedure, which depended on the physician’s procedural pace and the number of needle insertions; consequently, participants were exposed to the VR content for approximately 6 to 10 minutes. After the procedure, patients rested for approximately 15 minutes, after which the postprocedure questionnaires were administered. No participant requested discontinuation of the VR intervention or removed the headset before completion of the biopsy procedure, and all participants remained engaged with the video throughout the intervention.

For the control group, preprocedure questionnaires were administered 15 minutes before the biopsy procedure. No additional intervention was applied. The biopsy procedure lasted approximately 6 to 10 minutes. After the procedure, patients rested for approximately 15 minutes and then completed the postprocedure questionnaires.

### Nursing Intervention

Participants in the VR group received the intervention during the biopsy procedure. Before the procedure, patients completed the patient information form, VAS, BAI, and FPQ-III. During the biopsy, participants wore a VR headset displaying a motivational and relaxing underwater ocean video accompanied by music. The headset remained in place for approximately 6 to 10 minutes depending on the duration of the biopsy procedure. Before the biopsy, each participant was positioned according to the standard clinical protocol for thyroid FNAB, after which the VR headset was fitted while maintaining the required procedural position. Throughout the intervention, the researcher monitored participants to ensure that the headset remained comfortably positioned and that the video continued to play without interruption during the procedure.

After the procedure, patients rested for 15 minutes and then completed the VAS, BAI, and FPQ-III questionnaires again.

For the control group, the same preprocedure and postprocedure questionnaires were administered; however, no VR intervention was applied.

### Intervention Material

The VR intervention consisted of a relaxing motivational underwater ocean video (Figure 2 [25]) delivered through a VR SHINECON 3D VR headset compatible with 3.5- to 6.2-inch smartphones. The headset included adjustable lenses and an integrated audio system.

The content of the motivational video was determined based on expert opinions from 2 psychiatry faculty members, 1 psychiatry nurse, and 1 psychiatrist. The selection of the VR content was informed by previous literature demonstrating that VR-based distraction and nature-based virtual environments may reduce pain and anxiety during medical procedures [26,27]. Although the specific underwater video used in the present study had not been previously validated as a standardized intervention, it was selected through expert consensus to provide an immersive, calming, and emotionally neutral environment appropriate for patients undergoing thyroid biopsy. The experts evaluated the content for its suitability, absence of anxiety-provoking stimuli, and potential to facilitate attentional distraction during the procedure.

The selected video was streamed from YouTube and consisted of relaxing 360° underwater ocean scenes accompanied by background music [25]. All participants viewed the same video, which was started from the same point for each participant, and the audio volume was maintained at the same level for all participants. Although the video content and starting point were standardized, the duration of VR exposure varied between approximately 6 and 10 minutes according to the duration of the biopsy procedure. Head tracking was active in the 360° video mode, allowing the displayed field of view to change in response to participants’ head movements. However, because thyroid FNAB required participants to maintain a relatively stable head and neck position, active exploration of the 360° virtual environment through head movements was necessarily limited during the procedure.

The VR headset was disinfected with disinfectant solution after each use to ensure hygiene and patient safety (Figure 3). Throughout the VR intervention, participants were systematically monitored for potential adverse effects such as dizziness, nausea, eye strain, and discomfort in accordance with safety monitoring procedures. The protocol specified that the intervention would be discontinued if any adverse symptoms occurred, and clinical evaluation would be initiated when necessary.

**Figure 2:**
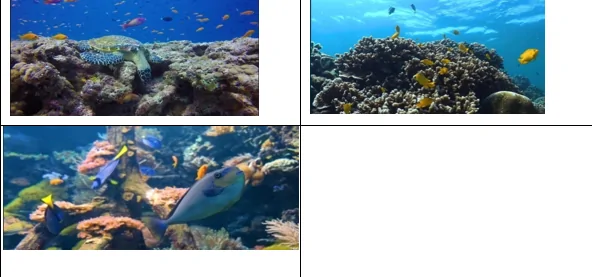
Examples of the motivational and relaxing video.

**Figure 3:**
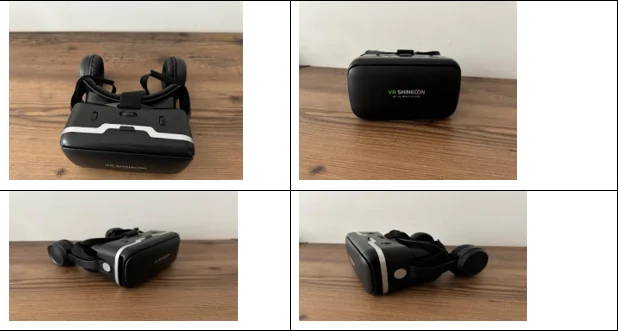
Virtual reality headset used in the study.

### Study Variables

Dependent variables included scores obtained from the VAS, BAI, and FPQ‑III. Independent variables included participants’ sociodemographic characteristics (age, gender, and education), smoking and alcohol use, exercise habits, medication use, history of thyroid nodules, family history of thyroid nodules, presence of neck swelling, swallowing or breathing difficulties, throat pressure sensation, voice changes, weight changes, fatigue, history of thyroid surgery, chronic disease, fear of FNAB, and prior experience with VR devices.

### Statistical Analysis

The data were analyzed using SPSS (version 26; IBM Corp). Descriptive statistics, including frequencies, percentages, means, and SDs, were used to summarize participants’ sociodemographic and clinical characteristics. Pearson chi-square test or Fisher exact test, as appropriate, was used to compare categorical variables between the VR and control groups. The Kolmogorov-Smirnov test was used to assess the normality of continuous variables. Because some continuous variables did not meet parametric assumptions, the Mann-Whitney *U* test was used for unadjusted between-group comparisons, and the Wilcoxon signed rank test was used for within-group pretest-to-posttest comparisons.

The primary analysis evaluated the effect of the intervention on the prespecified primary outcome, pain measured using the VAS, using ANCOVA, with the posttest VAS score as the dependent variable and adjustment for baseline VAS score and relevant baseline covariates. Secondary analyses evaluated the effects of the intervention on anxiety measured using the BAI and fear of pain measured using the FPQ-III, using the same ANCOVA approach with the corresponding posttest outcome as the dependent variable and its baseline score included as a covariate. Previous thyroid mass diagnosis and voice tone changes were included as additional covariates because they were considered clinically relevant baseline characteristics and showed an observed imbalance between the randomized groups. These covariates were not prespecified before analysis; therefore, the corresponding adjusted analyses should be interpreted with this consideration in mind.

For the ANCOVA models, adjusted group means (estimated marginal means) with 95% CIs, *F* test statistics, *P* values, and partial eta squared (η^2^) were reported. Homogeneity of variances was assessed using the Levene test. Unadjusted standardized between-group effect sizes (Cohen *d*) and their 95% CIs were calculated from the observed posttest group means and pooled SDs. These unadjusted Cohen *d* estimates were reported separately from the covariate-adjusted effect sizes (partial η^2^) obtained from the ANCOVA models. All statistical tests were 2-sided, and *P*<.05 was considered statistically significant.

During revision-stage data verification, we identified that a preliminary dataset containing 90 participants had inadvertently been used for the ANCOVA and effect-size calculations reported in the earlier version, instead of the final dataset containing all 100 randomized participants. The 10 omitted records were those entered after the preliminary dataset had been created and comprised 5 participants from the VR group and 5 from the control group. The error occurred because both the preliminary and final datasets had been retained in the analysis workflow, and the preliminary file was inadvertently selected when the subsequently requested ANCOVA and effect-size analyses were conducted. All inferential analyses, effect sizes, and CIs were subsequently recomputed using the verified complete dataset. The adjusted primary VAS result changed from not statistically significant (*P*=.15) to statistically significant (*P*=.048), changing the conclusion from no statistically significant between-group difference to a statistically significant difference favoring the VR group. Numerical estimates for the secondary outcomes, effect sizes, and CIs were also updated; however, their statistical significance did not change. The primary VAS outcome was the only outcome whose statistical significance status changed. All results reported in this article are based on the verified complete dataset.

### Ethical Considerations

Ethical approval for this study was granted by the Social and Human Sciences Ethics Committee of Dicle University on March 15, 2024 (approval number: 674771). Because the research intervention consisted of a nonpharmacological VR application and questionnaire-based assessments, while thyroid FNAB itself was performed as part of routine clinical care, no additional review by a clinical research ethics committee was sought. Institutional permission to conduct the study was obtained from Gaziantep City Hospital before data collection; this institutional permission did not constitute separate ethical approval. All procedures were conducted in accordance with the Declaration of Helsinki. Permission to use the BAI and FPQ-III was obtained from the respective rights holders.

All participants provided written informed consent before enrollment. Before providing consent, participants were informed about the study objectives, procedures, potential risks and benefits, the voluntary nature of participation, and their right to withdraw at any time without consequences.

Participant privacy and confidentiality were protected throughout the study. Direct identifiers were not included in the analytic dataset; study data were deidentified before analysis and stored in password-protected files accessible only to the research team. No identifiable participant information is reported in this manuscript.

Participants received no financial compensation, gifts, or other incentives for participation.

## Results

### Participant Characteristics

The study included 100 patients, with 50 allocated to the VR group and 50 to the control group. No adverse events related to the VR intervention, including dizziness, nausea, eye strain, or discomfort, were observed or reported during the study. No participant requested discontinuation of the VR intervention or removed the headset before completion of the biopsy procedure.

Of the 100 participants, 88 were female and 12 were male. The mean age was 47.51 (SD 12.83; range 21-80) years. Most participants were primary school graduates (n=73), 75 were nonsmokers, 63 reported regular medication use at home, and 8 reported engaging in regular exercise.

### Comparison of Sociodemographic Characteristics

The sociodemographic characteristics of participants in the VR and control groups are presented in Table 1. No statistically significant differences were found between the groups in terms of age, gender, educational status, smoking status, exercise habits, or regular medication use (*P*>.05). These findings indicate that the 2 groups were comparable and homogeneous in terms of sociodemographic characteristics (Table 1).

**Table 1:** Comparison of sociodemographic characteristics between the VR^a^ and control groups.

Variable			VR group (n=50)		Control group (n=50)		Test statistic		*P* value
Age (years), mean (SD)			48.84 (13.25)		46.18 (12.39)		—^b^		.35
Gender, n (%)							0.00 (1)^c^		.99
	Female	44 (88)		44 (88)					
	Male	6 (12)		6 (12)					
Education level, n (%)							0.38 (2)^c^		.82
	Primary school	36 (72)		37 (74)					
	High school	8 (16)		6 (12)					
	University	6 (12)		7 (14)					
Smoking status							2.61 (1)^c^		.10
	Yes	9 (18)		16 (32)					
	No	41 (82)		34 (68)					
Regular medication use							0.39 (1)^c^		.53
	Yes	33 (66)		30 (60)					
	No	17 (34)		20 (40)					
Exercise							—^d^		.71
	Yes	5 (10)		3 (6)					
	No	45 (90)		47 (94)					

^a^VR: virtual reality.

^b^The Mann-Whitney *U* test was used for comparison of continuous variables.

^c^The Chi-square test was used for comparison of categorical variables; test statistics are presented as chi-square (*df*).

^d^The 2-sided Fisher exact test was used when appropriate for comparison of categorical variables.

### Disease-Related Characteristics

The comparison of disease-related characteristics between the VR and control groups is provided in Table 2.

No statistically significant differences were found between the groups regarding family history of thyroid mass; sensation of thyroid enlargement or swelling; detection of swelling, nodule, or mass in the neck; difficulty swallowing or breathing; sensation of pressure in the throat; weight change; fatigue; presence of chronic disease; or fear of FNAB (*P*>.05).

However, a significant difference was observed between the groups regarding previous thyroid mass diagnosis and voice tone changes (*P*<.05). Participants in the VR group reported a higher prevalence of previous thyroid mass diagnosis (n=33, 66%) and voice tone changes (n=33, 66%) compared with the control group (Table 2).

As shown in Table 3, the mean VAS score decreased from 4.86 (SD 2.90) at pretest to 3.70 (SD 2.00) at posttest in the VR group, representing a statistically significant within-group reduction (Z=−2.868; *P*=.006). In the control group, the mean VAS score decreased slightly from 4.64 (SD 2.55) to 4.42 (SD 2.39); however, this change was not statistically significant (Z=−0.594; *P*=.55). Despite the significant within-group reduction observed in the VR group, the unadjusted between-group comparison of posttest VAS scores was not statistically significant (*P*=.12).

**Table 2:** Comparison of disease-related characteristics between the VR^a^ and control groups. The chi-square test was used for comparison of categorical variables.

Variable		VR group (n=50), n (%)	Control group (n=50), n (%)	Chi-square test (*df*)	*P* value	
Previous thyroid mass diagnosis				5.79 (1)		.01
	Yes	33 (66)	21 (42)			
	No	17 (34)	29 (58)			
Family history of thyroid mass				0.67 (1)		.41
	Yes	22 (44)	18 (36)			
	No	28 (56)	32 (64)			
Thyroid enlargement or swelling sensation				1.01 (1)		.31
	Yes	30 (60)	25 (50)			
	No	20 (40)	25 (50)			
Neck swelling or nodule detection				0.16 (1)		.68
	Yes	27 (54)	25 (50)			
	No	23 (46)	25 (50)			
Difficulty swallowing or breathing				0.04 (1)		.84
	Yes	27 (54)	26 (52)			
	No	23 (46)	24 (48)			
Pressure sensation in throat				0.37 (1)		.54
	Yes	30 (60)	27 (54)			
	No	20 (40)	23 (46)			
Voice tone change				5.80 (1)		.01
	Yes	33 (66)	21 (42)			
	No	17 (34)	29 (58)			
Weight change				1.00 (1)		.31
	Yes	23 (46)	28 (56)			
	No	27 (54)	22 (44)			
Fatigue or low energy				0.00 (1)		>.99
	Yes	42 (84)	42 (84)			
	No	8 (16)	8 (16)			
Chronic disease				1.48 (1)		.22
	Yes	24 (48)	18 (36)			
	No	26 (52)	32 (64)			
Fear of fine-needle aspiration biopsy				0.64 (1)		.42
	Yes	24 (48)	28 (56)			
	No	26 (52)	22 (44)			

^a^VR: virtual reality.

**Table 3:** Pretest and posttest scores and unadjusted comparisons of pain, anxiety, and fear of pain between and within groups. Within-group pretest-to-posttest comparisons were performed using the Wilcoxon signed rank test. Between-group *P* values represent unadjusted comparisons of posttest scores using the Mann-Whitney U test.

Outcome, questionnaire, and group					Pretest, mean (SD)	Posttest, mean (SD)	Within-group Z	Within-group *P* value	Between-group *P* value	
Primary outcome										
		VAS^a^								.12
				VR^b^	4.86 (2.90)	3.70 (2.00)	−2.868	.006		
				Control	4.64 (2.55)	4.42 (2.39)	−0.594	.55		
Secondary outcomes										
		BAI^c^								.55
				VR	19.48 (10.30)	9.14 (7.33)	−6.884	<.001		
				Control	16.70 (9.26)	10.50 (8.61)	−5.746	<.001		
		FPQ-III^d^								.26
				VR	59.98 (20.17)	52.84 (16.55)	−6.073	<.001		
				Control	58.02 (18.70)	54.60 (15.01)	−2.320	.03		

^a^VAS: visual analog scale.

^b^VR: virtual reality.

^c^BAI: Beck Anxiety Inventory.

^d^FPQ-III: Fear of Pain Questionnaire-III.

For the secondary outcomes, BAI scores decreased significantly from 19.48 (SD 10.30) to 9.14 (SD 7.33) in the VR group (Z=−6.884; *P*<.001) and from 16.70 (SD 9.26) to 10.50 (SD 8.61) in the control group (Z=−5.746; *P*<.001). Similarly, FPQ-III scores decreased significantly in both groups, from 59.98 (SD 20.17) to 52.84 (SD 16.55) in the VR group (Z=−6.073; *P*<.001) and from 58.02 (SD 18.70) to 54.60 (SD 15.01) in the control group (Z=−2.320; *P*=.02). However, the unadjusted between-group comparisons of posttest scores showed no statistically significant differences for either BAI (*P*=.55) or FPQ-III (*P*=.26; Table 3).

After adjustment for previous thyroid mass diagnosis, change in tone of voice, and the corresponding baseline outcome score, ANCOVA revealed a statistically significant between-group difference in posttest VAS scores, the primary outcome (*F*_1,95_=4.021; *P*=.048; partial η^2^=0.041). The adjusted mean VAS score was lower in the VR group (3.627, 95% CI 3.036-4.218) than in the control group (4.493, 95% CI 3.902-5.084).

For the secondary outcomes, no statistically significant adjusted between-group difference was observed in posttest BAI scores (*F*_1,95_=0.497; *P*=.48; partial η^2^=0.005). The adjusted mean BAI scores were 9.321 (95% CI 7.384-11.258) in the VR group and 10.319 (95% CI 8.382-12.256) in the control group. Similarly, the adjusted between-group difference in posttest FPQ-III scores did not reach statistical significance (*F*_1,95_=3.354; *P*=.07; partial η^2^=0.034), with adjusted means of 52.242 (95% CI 50.034-54.450) in the VR group and 55.198 (95% CI 52.990-57.406) in the control group (Table 4).

As shown in Table 5, the unadjusted standardized between-group effect size for the primary outcome, posttest VAS score, was small (Cohen *d*=−0.33, 95% CI −0.72 to 0.07), indicating lower observed pain scores in the VR group than in the control group. For the secondary outcomes, the effect sizes were also small for posttest BAI (Cohen *d*=−0.17, 95% CI −0.56 to 0.22) and FPQ-III scores (Cohen *d*=−0.11, 95% CI −0.50 to 0.28).

**Table 4:** Adjusted comparisons of posttest pain, anxiety, and fear of pain scores between the VR^a^ and control groups based on ANCOVA^b^. Values are estimated marginal means (adjusted means) with 95% CIs. ANCOVA compared posttest outcomes between the VR and control groups after adjustment for previous thyroid mass diagnosis, change in tone of voice, and the corresponding baseline outcome score. Partial η2 represents the effect size for the adjusted group effect.

Outcome and posttest score			VR group, adjusted mean (95% CI)		Control group, adjusted mean (95% CI)		Covariates		*F* test (*df*); *P* value; partial η^2^
Primary outcome									
	VAS^c^	3.627 (3.036-4.218)		4.493 (3.902-5.084)		Previous thyroid mass diagnosis, change in tone of voice, and baseline VAS score		4.021 (1, 95); .048; 0.041	
Secondary outcomes									
	BAI^d^	9.321 (7.384-11.258)		10.319 (8.382-12.256)		Previous thyroid mass diagnosis, change in tone of voice, and baseline BAI score		0.497 (1, 95); .48; 0.005	
	FPQ-III^e^	52.242 (50.034-54.450)		55.198 (52.990-57.406)		Previous thyroid mass diagnosis, change in tone of voice, and baseline FPQ-III score		3.354 (1, 95); .07; 0.034	

^a^VR: virtual reality.

^b^ANCOVA: analysis of covariance.

^c^VAS: visual analog scale.

^d^BAI: Beck Anxiety Inventory.

^e^FPQ-III: Fear of Pain Questionnaire-III.

**Table 5:** Unadjusted standardized effect sizes (Cohen d) and 95% CIs for posttest outcomes. Cohen d values represent unadjusted standardized between-group differences based on observed posttest scores and were calculated using the pooled SD. Negative values indicate lower posttest scores in the VR^a^ group than in the control group. Cohen d values should be distinguished from the covariate-adjusted effect sizes (partial η2) obtained from the ANCOVA^b^ models presented in Table 4.

Outcome and posttest score		VR group, mean (SD)	Control group, mean (SD)	Cohen *d* (95% CI)
Primary outcome				
	VAS^c^	3.70 (2.00)	4.42 (2.39)	−0.33 (−0.72 to 0.07)
Secondary outcomes				
	BAI^d^	9.14 (7.34)	10.50 (8.61)	−0.17 (−0.56 to 0.22)
	FPQ-III^e^	52.84 (16.55)	54.60 (15.01)	−0.11 (−0.50 to 0.28)

^a^VR: virtual reality.

^b^ANCOVA: analysis of covariance.

^c^VAS: visual analog scale.

^d^BAI: Beck Anxiety Inventory.

^e^FPQ-III: Fear of Pain Questionnaire-III.

## Discussion

### Principal Findings

This open-label randomized controlled trial evaluated the effects of a VR intervention on pain, anxiety, and fear of pain in patients undergoing thyroid FNAB. After adjustment for baseline outcome values and relevant clinical covariates, the VR group had significantly lower postprocedural pain scores than the control group, supporting the primary hypothesis. The magnitude of the adjusted group effect on pain was small. In contrast, adjusted between-group differences in anxiety and fear of pain did not reach statistical significance. Nevertheless, the within-group findings and the direction of the adjusted estimates, particularly for fear of pain, suggest that the effects of VR may differ across procedural and psychological outcomes.

### Findings Related to Pain

For the primary outcome, postprocedural pain was significantly lower in the VR group than in the control group after adjustment for baseline pain, previous thyroid mass diagnosis, and voice tone changes. This finding supports the primary study hypothesis and suggests that VR may provide an additional analgesic benefit during thyroid FNAB. Consistent with the adjusted analysis, pain decreased significantly from pretest to posttest in the VR group, whereas no significant within-group change was observed in the control group.

This finding is also supported by emerging procedure-specific evidence. Schaake et al [28], in a randomized controlled trial involving patients undergoing thyroid FNAB, reported a significantly greater reduction in pain among patients receiving VR in addition to standard care than among those receiving standard care alone. Although the intervention and analytical approach differed from those used in the present study, the consistent direction of the effect provides direct support for the potential analgesic benefit of VR during thyroid FNAB. Evidence from other biopsy procedures also supports this finding. In a systematic review and meta-analysis involving 445 adults undergoing puncture biopsy procedures, VR significantly reduced pain compared with control conditions, although substantial heterogeneity was observed across studies and the certainty of evidence was rated as low [29]. Similarly, another systematic review and meta-analysis found significant reductions in both intraprocedural and postprocedural pain with VR compared with standard care [30].

Previous studies involving needle-related and other painful clinical procedures have also demonstrated beneficial effects of VR on procedural pain [15,31-37]. These effects are commonly explained by attentional distraction mechanisms, whereby immersive audiovisual stimuli may redirect limited attentional resources away from nociceptive input, thereby reducing the cognitive processing and perceived intensity of painful stimuli [15,31-37]. Although this mechanism was not directly evaluated in the present study, it may provide a plausible explanation for the greater reduction in pain observed in the VR group.

### Findings Related to Anxiety

For the secondary outcome of anxiety, no statistically significant difference was observed between the VR and control groups in postintervention BAI scores after adjustment for baseline covariates. Although anxiety decreased over time in both groups in the within-group analyses, these reductions did not result in a significant between-group difference, suggesting that VR did not provide a detectable additional anxiolytic effect during thyroid FNAB in the present study.

Previous research has demonstrated beneficial effects of VR on procedural anxiety in different clinical contexts, including anxiety-provoking needle-related procedures [38,39]. However, evidence directly related to thyroid biopsy is consistent with the present findings. Schaake et al [28], in a randomized controlled trial involving patients undergoing thyroid FNAB, found no statistically significant difference in the change in anxiety between the VR plus standard-care and standard-care groups. Notably, anxiety in that study was assessed using a VAS rather than the BAI used in the present study. Thus, despite the use of different anxiety measures, both studies found no significant additional effect of VR on anxiety during thyroid biopsy.

Evidence from the broader literature also suggests that the anxiolytic effects of VR may vary according to procedural context and the timing of anxiety assessment. Kodvavi et al [30], in a systematic review and meta-analysis of randomized controlled trials involving adults undergoing elective medical procedures, found that VR significantly reduced postprocedural anxiety but had no significant effect on preprocedural anxiety. These findings indicate that the effects of VR on anxiety may not be uniform across different procedural settings and phases. In the present study, the substantial reduction in anxiety observed in both groups may similarly have reflected factors common to the biopsy experience, including completion of the procedure and the associated reduction in procedure-related anticipation and uncertainty, thereby potentially limiting the ability to detect an additional anxiolytic effect specifically attributable to VR. Accordingly, although VR has demonstrated beneficial effects on anxiety in other procedural settings [38-40], its additional effect on anxiety during a brief procedure such as thyroid FNAB may be less pronounced.

### Findings Related to Fear of Pain

For the secondary outcome of fear of pain, no statistically significant between-group difference was observed in posttest FPQ-III scores after adjustment for baseline FPQ-III score, previous thyroid mass diagnosis, and change in tone of voice (*F*_1,95_=3.354; *P*=.07; partial η^2^=0.034). Although FPQ-III scores decreased over time in both groups, these within-group changes should not be interpreted as evidence of a VR-specific effect because the adjusted between-group comparison did not reach statistical significance.

Fear experienced during thyroid FNAB may reflect not only the anticipated physical discomfort associated with needle insertion but also factors such as expectations of pain and diagnostic uncertainty. Şenoymak et al [10] reported that fear of pain and concern about a possible malignant diagnosis were important sources of preprocedural anxiety among patients undergoing thyroid FNAB. Similarly, fear of pain and procedural factors such as the number of biopsied nodules have been associated with patients’ pain and fear responses [41]. These findings suggest that fear of pain in the context of thyroid FNAB may represent a multifactorial experience.

Studies using the FPQ-III have shown that fear of pain is associated with pain experiences and pain-related outcomes in surgical patients [42-44]. In addition, Çalışkan et al [45] reported that education addressing pain and the surgical process could reduce fear of medical pain assessed using the FPQ-III. In contrast, the VR intervention in the present study primarily provided immersive audiovisual distraction during the procedure rather than directly targeting pain-related expectations or concerns [45]. This difference in intervention focus may partly explain why VR significantly reduced pain in the present study but did not produce a statistically significant independent effect on fear of pain. Although previous studies support the potential of VR to reduce procedural distress, pain, anxiety, or fear [36-50], more directly targeted interventions may be needed to influence a complex cognitive and anticipatory outcome such as fear of pain.

### Covariate Effects and Baseline Imbalances

The ANCOVA models indicated that baseline outcome measures were important predictors of postintervention scores across the assessed domains. Importantly, after adjustment for these baseline measures and the imbalanced clinical characteristics, group allocation remained significantly associated with postprocedural pain, whereas the adjusted group effects for anxiety and fear of pain did not reach statistical significance.

Taken together, these findings support the primary hypothesis that VR reduces procedural pain during thyroid FNAB, although the magnitude of the effect was small. In contrast, the secondary hypotheses concerning anxiety and fear of pain were not supported by the adjusted between-group analyses.

### Limitations

This study has several limitations that should be considered when interpreting the findings. First, because of the nature of the VR intervention, blinding of participants and health care providers was not feasible. Participants and health care providers were aware of the intervention condition, which may have introduced performance and expectancy bias. In addition, although participants were assigned according to a preprepared randomization list, no formal allocation concealment mechanism (eg, sequentially numbered opaque sealed envelopes or centralized allocation) was used. Therefore, the possibility of selection bias cannot be completely excluded.

Second, the study was conducted in a single tertiary care center, and the sample was predominantly female (88/100, 88%) and consisted largely of participants with a primary school education (73/100, 73%). Although the predominance of women is consistent with the higher prevalence of thyroid nodules among women, the sex and educational composition of the sample may limit the generalizability of the findings to populations with different demographic and educational characteristics.

Third, although baseline differences between groups in certain clinical variables (eg, previous thyroid mass diagnosis and voice tone changes) were adjusted using ANCOVA, residual confounding due to unmeasured or unknown variables cannot be completely excluded. Therefore, despite statistical adjustment, the possibility that unmeasured confounders influenced the observed outcomes should be considered when interpreting the findings.

Fourth, the VR intervention was based on preselected passive audiovisual content rather than a fully standardized interactive therapeutic protocol. Although this approach ensured consistency across participants, participant engagement and immersion may have varied, potentially reducing the effectiveness of the intervention and limiting the reproducibility of the findings. More interactive or personalized VR environments may produce greater levels of engagement and clinical benefit. Future studies should compare passive and interactive VR applications to determine whether the level of immersion influences pain, anxiety, and fear during thyroid FNAB.

Fifth, the study evaluated only the short-term effects of the intervention following the biopsy procedure. Therefore, the longer-term effects of VR on pain, anxiety, and fear were not assessed, limiting conclusions regarding the sustainability of its potential benefits. In addition, the temporal characteristics of the secondary outcome measures may have limited their responsiveness over the short assessment interval. Specifically, the BAI’s past-week recall period overlapped almost entirely between the preprocedure and postprocedure assessments, while the FPQ-III assesses fear of pain across hypothetical scenarios and may reflect a relatively trait-like construct. These features may have reduced sensitivity to immediate procedure-related changes over the approximately 30-minute assessment interval and provide an alternative explanation for the null secondary findings.

Finally, the sensitivity analysis indicated that the final sample provided 80% power to detect an ANCOVA effect size of approximately *f*=0.283 or larger; therefore, the study had limited sensitivity for smaller effects. Although the adjusted between-group difference in the primary outcome (VAS) reached statistical significance, the effect magnitude was small (partial η^2^=.041), and the corresponding unadjusted standardized effect estimate was also small (Cohen *d*=−0.33, 95% CI −0.72 to 0.07). Accordingly, the statistically significant adjusted finding should be interpreted cautiously, and larger adequately powered trials are needed to establish the magnitude and clinical relevance of the effect.

### Conclusions

This randomized controlled trial demonstrated that VR used during thyroid FNAB resulted in significantly lower postprocedural pain than standard care after adjustment for baseline pain and relevant clinical covariates. However, the magnitude of the adjusted effect was small, and no significant between-group effects were identified for anxiety or fear of pain. These findings suggest that VR may provide a modest nonpharmacological analgesic benefit during thyroid FNAB, but its effects may vary according to the outcome assessed and should not be generalized to all dimensions of procedural distress.

As a low-burden and noninvasive intervention, VR may be considered an adjunct to standard nursing care, particularly for patients who are concerned about procedural pain or who prefer nonpharmacological support. Nevertheless, VR should complement rather than replace person-centered communication, procedural information, reassurance, and other strategies addressing the cognitive and emotional components of anxiety and fear. Its routine clinical use cannot yet be recommended solely on the basis of the present findings because the observed pain reduction was small and its clinical importance remains uncertain.

Larger, adequately powered multicenter trials are needed to confirm the analgesic effect and determine whether it represents a clinically meaningful improvement. Future studies should identify the patients most likely to benefit, use standardized intervention protocols, and compare passive VR content with more interactive or personalized environments. Research should also examine whether combining VR with preparatory education, guided breathing, or other nursing interventions produces broader effects on pain, anxiety, and fear of pain during thyroid FNAB and comparable brief needle-based procedures.

## Appendix

Multimedia Appendix 1 CONSORT-EHEALTH checklist.

## Abbreviations

ANCOVA: analysis of covariance
BAI: Beck Anxiety Inventory
CONSORT: Consolidated Standards of Reporting Trials
CONSORT-EHEALTH: Consolidated Standards of Reporting Trials of Electronic and Mobile Health Applications and Online Telehealth
FNAB: fine-needle aspiration biopsy
FPQ-III: Fear of Pain Questionnaire-III
VAS: visual analog scale
VR: virtual reality

## Data Availability

The datasets generated or analyzed during this study are available from the corresponding author on reasonable request.
