# Supplementary material for: Effects of Virtual Reality on Pain, Anxiety, and Fear During Thyroid Fine-Needle Aspiration Biopsy: Open-Label Randomized Controlled Trial
Source: J Med Internet Res. 2026 Oct 6;28:e103829. doi: 10.2196/103829 (PMC13684117; doi:10.2196/103829)
Supplement: Supplementary file 1 [file jmir_v28i1e103829_app1.pdf]

CONSORT-EHEALTH Checklist

Effectsof Virtual Reality on Pain, Anxiety,and Fear During Thyroid Fine-Needle Aspiration Biopsy: An Open-Label Randomized Controlled Trial

Updated against the revised manuscript (August2026)

TITLE AND ABSTRACT

Item	Description	Locationinrevisedmanuscript
1a-i	Identification of the mode of delivery (virtualreality) in the title	Page1(Titlepage)
1a-iii	Identification of the target population in the title	Page1(Titlepage)
1b-i	Key features and components of the VR intervention described in the abstract	Page2(Abstract:Methods)
1b-ii	Level of human involvement described	Page2(Abstract:Methods)
1b-iii	Recruitment and assessment procedures summarized	Page2(Abstract:Methods)
1b-iv	Numbersr and omized and analyzed reported	Page2(Abstract:MethodsandResults)

INTRODUCTION

Item	Description	Locationinrevisedmanuscript
2a-i	Clinical problem and rationale for VR as apotential solution	Pages3-5(Introduction)
2a-ii	Scientific rationale and prior evidence for VR-based pain and anxiety management	Pages3-5(Introduction)
2b	Study objective and prespecified primary and secondary hypotheses	Page5(ObjectiveandResearch Hypotheses)

METHODS - TRIAL DESIGN AND PARTICIPANTS

Item	Description	Locationinrevisedmanuscript
3a	Randomized controlled trial design, including open-label status and blinded statistical analysis	Page 7 (StudyDesign)
3b	Changes to the intervention or procedure saftertrialcommencement	Page 12: pilot testing completed before the main study; no protocol modifications were required
4a-i	Eligibilitycriteria, including noprevious VR experience	Page8(InclusionandExclusionCriteria)
4a-ii	Recruitmentsetting,recruitmentframework,andface-to-faceassessment	Pages7-8(SettingandTime;Population and Sample)
4a-iii	Written informed consent procedures	Page18(EthicalConsiderations)
4b-i	How data were collected and assessments administered	Pages12-13(DataCollectionProcedure)

METHODS - INTERVENTION

Item	Description	Locationinrevisedmanuscript
5-i	Intervention hardware and content source/owner ship information	Pages13-14(InterventionMaterial);

		Reference 27
5-ii	Development, expertreview, and formative/pilot evaluation	Pages 12-14 (pilot testing and expert-informedcontentselection)
5-iii	Intervention version/date or stable content identification	Pages 13-14; standardized YouTube video identified by URL in Reference 27
5-v	Screen shots or visual representation of the intervention	Figures2-3,Page14-15
5-vii	Access to the intervention and delivery context	Pages14-15(InterventionMaterial)
5-viii	Detailed description of VR hardware, audiovisual content, head tracking, and standardization	Pages14-15(InterventionMaterial)
Item	Description	Locationinrevisedmanuscript
5-ix	Intended exposure/dose and duration	Pages12-13:approximately6-10minutes during FNAB
5-x	Human involvement and monitoring during the intervention	Pages 11-14 (Data Collection Procedure; Nursing Intervention; Intervention Material)
5-xi	Promptsor reminders	Notapplicable
5-xii	Co-interventions, positioning, support,and standard care	Pages12-14(NursingInterventionand control condition)

METHODS - OUTCOMES, SAMPLE SIZE, RANDOMIZATION, AND BLINDING

Item	Description	Locationinrevisedmanuscript
6a-i	Primary and secondary outcomes and measurement tools	Pages11-12(DataCollectionTools)
6a-ii	Use of and exposure to the VR intervention	Pages13-14
6a-iii	Qualitative feedback	Notcollected/reported
7a-i	Pre-determined sample size calculation and ANCOVA sensitivity analysis	Page8(SampleSizeandPowerAnalysis)
8a	Random sequence generation	Pages9-10(RandomizationandBlinding)
8b	Randomization type: sex-stratified assignment	Pages9-10(RandomizationandBlinding)
9	Allocation concealment mechanism	Page8:noformalallocationconcealment mechanism was used
10	Implementation of enrollment and group assignment	Pages9-10(RandomizationandBlinding)
11a-i	Blinding: open-label study; independent statistician blinded to allocation	Pages 9 and 10
11a-ii	Information provided during consent and assignment	Pages 11 and 15
11b	Similarity of interventions	Notapplicable;controlgroupreceived standard care without VR

METHODS - STATISTICS AND ETHICS

Item	Description	Locationinrevisedmanuscript

12a-i	Handling of missing data and attrition	Pages 10 and 16-17:nopostrandomization attrition; all 100 randomized participants were analyzed
12b	Additional analyses, adjusted models, and effect size reporting	Pages 17-18 (StatisticalAnalysis) and<br>Pages 22-24 (Tables 3-5)
X26-i	Research ethics approval and institutional permission	Page 18 (EthicalConsiderations)
X26-ii	Written informed consent and the right to withdraw	Page 18 (EthicalConsiderations)
X26-iii	Safetymonitoring,privacy,confidentiality,de-identification,andsecurestorage	Pages 15-16

RESULTS

Item	Description	Locationinrevisedmanuscript
13a	Participant flow	Figure 1,Page 10
13b-i	Exclusions and attrition	Figure 1, Page10; Results, Page19
14a	Recruitment dates	Page 2 (Abstract) and Page 7 (Setting and Time)
14b	Trial completion	Page 7;completed April 30, 2025
15-i	Baseline demographic and clinical characteristics	Tables 1-2, Pages 19-20
16-i	Number analyzed in each group	Pages19-24: VR n=50;controln=50
16-ii	Analysis population	Pages19-24:all randomized participants were included in the reported analyses
Item	Description	Locationinrevisedmanuscript
17a-i	Primary and secondary clinical outcomes and intervention exposure	Pages 22-24 (Tables 3-5 and accompanying text)
17b	Adjusted and unadjusted effect estimates with confidence intervals/effect sizes	Pages23-24 (Tables4-5)
18-i	Ancillary and within-group analyses	Pages23 (Table3 andaccompanying text)
19-i	Monitoring for dizziness, nausea, eyestrain, and discomfort	Page 14 (Intervention Material) and<br>Page 19 (Results)
19-ii	Unintended effects, discontinuation, and participant engagement	Pages 13 and 19: no adverse events or<br>VR discontinuations

DISCUSSION

Item	Description	Locationinrevisedmanuscript
22-i	Interpretation of the primary and secondary outcomes	Pages 25-28 (Discussion)
22-ii	Implications and directions for future research	Pages 25-29 (Discussion and Conclusions)
20-i	Potential biases and limitations	Pages 29-31 (Limitations)
21-i	Generalisability to broader populations	Pages 29-32 (Limitations and Conclusions)

21-ii	Applicability outside the trial setting	Pages 29-32 (Limitations and Conclusions)

OTHER INFORMATION

Item	Description	Locationinrevisedmanuscript
23	Trial registration	Page2; Page8; Declarations, Page 33: ClinicalTrials.gov NCT06792929
24	Protocol availability	Trial registration cited in Methods and Declarations;aseparateprotocol-access statement is not provided
25	Funding	Declarations, Page33:no study funding; APC support acknowledged separately
X27	Conflict Of Interest And Relationship to the intervention	Declarations, Page 33:no competing interests

Note: Page locations reflect the pagination of the revised manuscript supplied for this checklist update. Figure, table, section, and reference identifiers are included to facilitate verification if the submission system generates different PDF pagination.
